# The genomic prehistory of peoples speaking Khoisan languages

**DOI:** 10.1093/hmg/ddaa221

**Published:** 2020-10-19

**Authors:** Brigitte Pakendorf, Mark Stoneking

**Affiliations:** Dynamique du Langage, UMR5596, CNRS & Université de Lyon, 14 avenue Berthelot, 69007 Lyon, France; Department of Evolutionary Genetics, MPI for Evolutionary Anthropology, Deutscher Platz 6, 04103 Leipzig, Germany

## Abstract

Peoples speaking so-called Khoisan languages—that is, indigenous languages of southern Africa that do not belong to the Bantu family—are culturally and linguistically diverse. They comprise herders, hunter-gatherers as well as groups of mixed modes of subsistence, and their languages are classified into three distinct language families. This cultural and linguistic variation is mirrored by extensive genetic diversity. We here review the recent genomics literature and discuss the genetic evidence for a formerly wider geographic spread of peoples with Khoisan-related ancestry, for the deep divergence among populations speaking Khoisan languages overlaid by more recent gene flow among these groups and for the impact of admixture with immigrant food-producers in their prehistory.

## Introduction

In this paper, we use the term ‘Khoisan’ as a loose cover term to refer to the indigenous languages of southern Africa that do not belong to the Bantu family—and that are most saliently characterized by their heavy use of click consonants—as well as by extension to the genetic ancestry associated with the peoples who speak these languages. The term was coined by the biological anthropologist Schulze in 1928 by combining the Khoekhoe herders’ term for themselves with their term for foragers (1); variations encountered in the literature are Khoe-San and KhoeSan. Given the fact that the peoples speaking Khoisan languages are culturally and linguistically distinct and each has their own particular history, all umbrella terms are flawed; it is thus of crucial importance to keep in mind that use of a single term does not signify a unified entity.

**Figure 1:**
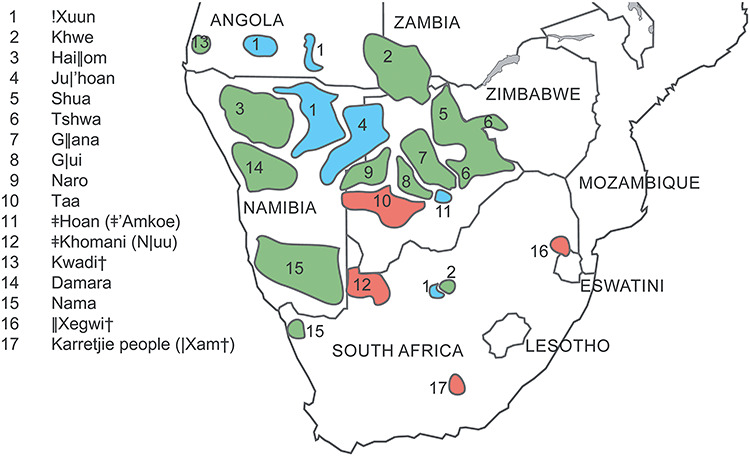
Map showing the approximate location of Khoisan-speaking groups, based on ethnolinguistic data from Güldemann (1) and Hitchcock (2), and information on the Karretjie from Schlebusch *et al*. (52). Colours indicate the language family affiliation: blue, Kx’a; red, Tuu; green, Khoe–Kwadi. Languages that are extinct are indicated by crosses. Some of the ǂKhomani still remember N|uu. ǂ’Amkoe is the actual name of the language spoken by the ǂHoan, but as the initial publication presenting genetic data from this group referred to them by the old language name, this has been maintained in genetic publications.

Nowadays, peoples speaking Khoisan languages live mainly in Namibia, Botswana and Angola, with smaller numbers in Zambia, Zimbabwe, South Africa, Lesotho and Eswatini (2) (Fig. 1). In South Africa, most historically known Khoisan groups have given up their original languages and have merged with so-called Coloured populations (3). Two groups often included in recent genetic studies are the Karretjie people, itinerant sheepshearers of the Karoo who are probably partly descendants of |Xam hunter-gatherers (4), and the ǂKhomani. This latter is a group of people with diverse Khoisan-related ancestries tracing back to the southern Kalahari, who joined together in the 1990s in order to file a land rights claim (5).

Although an initial broad classification of the languages of Africa identified a single Khoisan phylum comprising three branches in southern Africa plus two languages—Sandawe and Hadza—spoken in East Africa (6), nowadays specialists of these languages agree that there are three distinct language families in southern Africa, namely Kx’a, Tuu and Khoe–Kwadi (1). Of these, Kx’a and Tuu might ultimately descend from a shared ancestor, but that has not yet been conclusively demonstrated (7). The Khoe–Kwadi languages are not related to either the Kx’a or the Tuu languages (1,8). As for the East African languages, although there is no demonstrable relationship between Hadza and any of the southern African Khoisan languages, there is some indication that Sandawe might be related to the Khoe–Kwadi family; however, this, too, needs further corroboration (7).

Culturally, too, there is considerable heterogeneity among the Khoisan-speaking peoples of southern Africa (9): herding groups are known historically from coastal and interior regions of the Cape, the descendants of whom are the Nama (nowadays settled mainly in southern Namibia) as well as several Coloured groups in South Africa (9,10). Furthermore, the Kwepe, small-stock pastoralists from southwestern Angola, are known to have spoken Kwadi, a language of the Khoe–Kwadi family, although this is nowadays practically extinct (9,11). Hunter-gatherers roamed the Cape interior of South Africa in historic times and are still found in the Kalahari region spanning Namibia, Botswana and parts of South Africa. But there are also groups that do not neatly fit into the herder–forager dichotomy. Foremost among these are the Damara, a peripatetic group (12) who traditionally practiced foraging, small-scale herding of goats and blacksmithing in a client relationship to the Nama and the Bantu-speaking pastoralist Herero. Along the Kavango River, the Khwe rely on fishing as well as hunting and gathering, whereas in the eastern Kalahari, the Shua and Tshwa are transitioning to food production and are in a client relationship to their Bantu neighbours in addition to their foraging subsistence.

Given this linguistic and cultural diversity, it is clear that the prehistory of the peoples speaking Khoisan languages must have been highly complex. Numerous studies over the past decade have highlighted the considerable genetic diversity found in these groups, the deep divergence among them as well as between them and other African groups, and the impact of successive waves of migration of food-producing peoples from East and West–Central Africa as well as in historical times of European colonizers [see (13) for a recent review]. We here survey the recent literature and discuss the genetic evidence for an erstwhile wider geographic spread of peoples with Khoisan-related ancestry, for the deep divergence among populations speaking Khoisan languages overlaid by more recent gene flow among these groups and for the impact of admixture with immigrant food-producers in their prehistory. For convenience, we will refer to peoples speaking a Khoisan language as a Khoisan-speaking group and to peoples speaking a Bantu language as a Bantu-speaking group (although of course each group speaks a particular language belonging to the Kx’a, Tuu or Khoe–Kwadi families for Khoisan-speaking groups, or to the Bantu family). Throughout the paper, we follow the nomenclature of Güldemann (1), irrespective of the spelling of group names found in individual articles.

## A Wider Geographic Spread in Prehistoric Times

Recent genome-wide analyses of DNA from ancient human remains in East Africa have demonstrated the presence in the past of Khoisan-related ancestry in regions as distant from the Kalahari as Tanzania and Kenya (throughout this review, for convenience, we use present-day countries to refer to the location of ancient remains that predate country formation). Thus, ~60% of the ancestry of ancient remains from Malawi dated to between 2500 and 8100 BP and ~30% of the ancestry of a 1400-year-old individual from Tanzania is related to ancestry detectable both in 2000-year-old hunter-gatherer remains from South Africa and modern Ju|’hoan (14). Similarly, an ancient individual from Kenya dated to 3500 BP shows evidence of low levels of Khoisan-related ancestry (15). In addition, there is evidence from whole genome sequences from modern populations for potential long-distance migrations involving Khoisan-speaking groups, e.g. in the sharing of private alleles between the Ju|’hoan and Mbuti central African rain forest foragers (16).

**Table 1:** Salient features of recent studies of whole genome sequences that included Khoisan-speaking groups

Study	Sample sizes and groups	Deepest divergence time between Khoisan-speaking and other groups	Divergence time among Khoisan-speaking groups
Fan *et al*. (19)	Four Ju|’hoan^a^ Two ǂKhomani^a^	~200 kya	~30 kya
Lorente-Galdos *et al*. (20)	Two Ju|’hoan^b^ One Taa^c^ One ǂKhomani	~190 kya	n/a
Bergström *et al*. (21)	Six Ju|’hoan^d^	~162 kya	n/a
Schlebusch *et al*. (16)	Five Ju|’hoan Five ǂKhomani Five Nama Five Karretjie Five G|ui/G‖ana^e^	~200–300 kya	~160–190 kya

^a^Sequences from Mallick *et al*. (53); Ju|’hoan samples from the Human Genome Diversity Panel (HGDP).

^b^One sequence from Mallick *et al*. (53) and one sequence from Meyer *et al*. (54); samples from HGDP.

^c^Sequence from Schuster *et al*. (55), where this individual (KB1) is labelled a Tuu speaker.

^d^All samples from HGDP and include the four Ju|’hoan from Mallick *et al*. (53).

^e^Mixed group of G|ui and G‖ana individuals, see Schlebusch *et al*. (30) for details.

Interestingly, the Khoisan-related ancestry in eastern Africa is related in equal degrees to the deeply diverging lineages identified in modern-day Khoisan-speaking populations see ``High Levels of Genetic Diversity in Khoisan-Speaking Peoples'', implying that the Khoisan-related groups settled in eastern Africa were genetically distinct from those living in southern Africa (14). These results mirror the results of mitochondrial DNA (mtDNA) analyses that found a complementary distribution of one of the Khoisan-specific haplogroups, L0k. Of three deeply divergent branches (L0k1a, L0k1b and L0k2), only L0k1a is found among extant Khoisan-speaking groups of Namibia and Botswana, whereas L0k1b and L0k2 are found practically exclusively in Bantu-speaking populations settled in Zambia. This implies that people genetically related to currently known Khoisan-speaking groups, yet carrying distinct lineages, were resident in regions beyond those previously attested (17). There are no historically known Khoisan-speaking groups in either Zambia or Malawi or further northeast, and modern-day populations of Malawi show no traces of Khoisan-related ancestry. It is thus clear that the incoming Bantu-speaking populations must have replaced the Khoisan-related autochthonous populations with hardly any admixture. Linguistic analyses, too, show that some Bantu languages of the Kavango–Zambezi transfrontier area borrowed words with click consonants from Khoisan languages that are nowadays extinct, in addition to borrowing words from Khwe and Ju languages (18).

## High Levels of Genetic Diversity in Khoisan-Speaking Peoples

Khoisan-speaking groups are consistently found to harbour high levels of genetic diversity. Several recent studies of whole genome sequences found highest levels of genetic diversity in Khoisan-speaking individuals (16,19–21), and these individuals also have the highest frequencies, on average, of population-specific copy number variants worldwide (22). However, sample sizes and ethnolinguistic diversity of the groups analyzed remain quite limited (Table 1); there is a clear need for additional whole genome sequence studies of further Khoisan-speaking groups.

Khoisan-speaking groups are also the first to branch off in genomic studies of African or world-wide populations (16,19,20,23), with their divergence from other populations dated to 160–300 kya (Table 1). The fact that Khoisan-related lineages are the first to diverge has sometimes been erroneously interpreted as strong evidence for an origin of modern humans in southern Africa [(24), which is based solely on mtDNA lineages; see (25,26) for substantial critiques of this paper]. However, as noted above, Khoisan-related groups were formerly more widespread, and moreover, the divergence between Khoisan-speaking groups and other African groups could, in principle, have occurred anywhere in Africa.

Khoisan-speaking peoples also show evidence of a larger effective population size over time than other African populations (16,19,20,27). All human populations show a signal of decreasing effective population size beginning around the time of the divergence of African from non-African populations, ~50–100 kya; however, Khoisan-speaking groups show less of a reduction in effective population size than do other populations (16,19,20). Some of this diversity might be due to archaic admixture from an as yet undiscovered population (28). For example, whole genome sequencing (20) suggests ~4% ancestry from an archaic ‘ghost’ population in the four Khoisan-speaking individuals analyzed.

**Figure 2:**
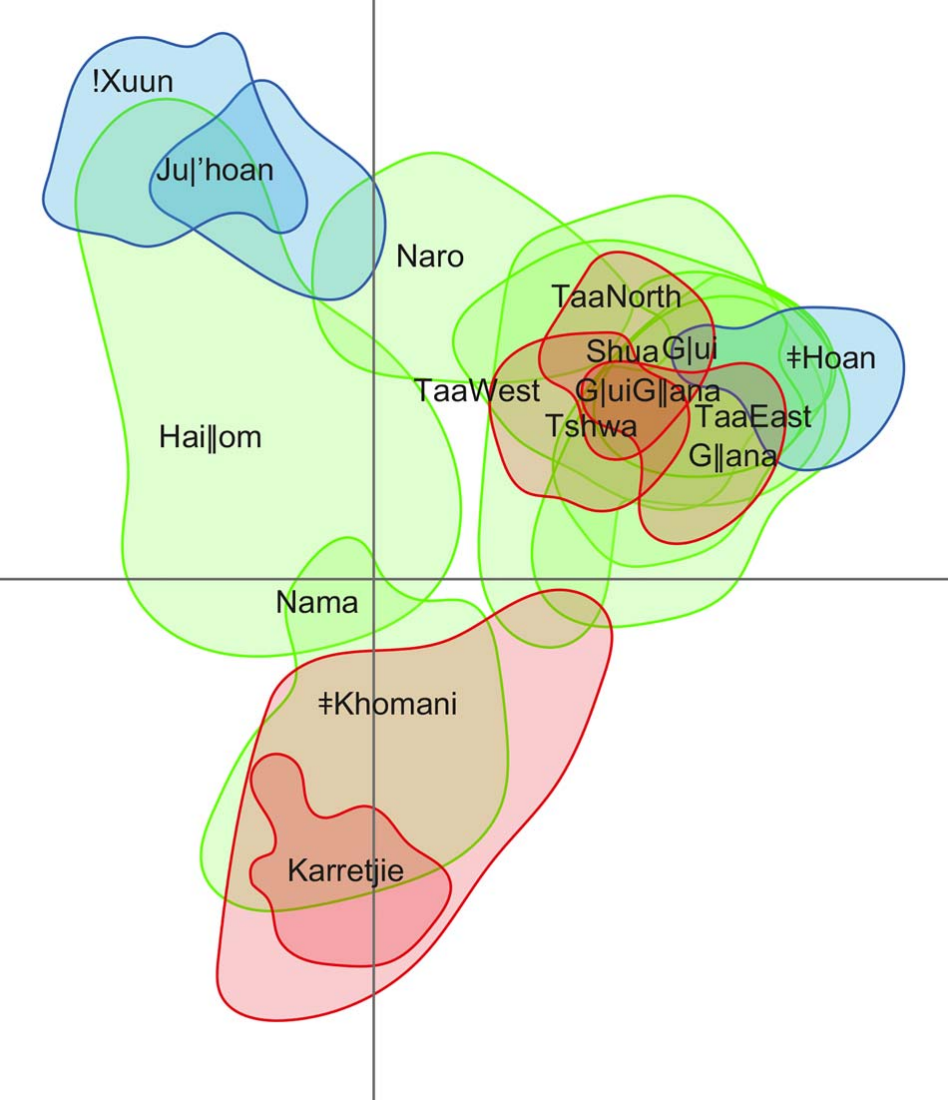
Plot of the first two dimensions of a multidimensional scaling analysis of Khoisan-speaking groups, illustrating the northern, central and southern genetic groupings of Khoisan-speaking groups. The plot is based on genome-wide SNP array data with non-Khoisan-related ancestry masked. The vertical axis is the first dimension and the horizontal axis is the second dimension. The contours depict 90% utilization distribution densities, i.e. the smallest area of the plot in which there is a 90% probability of locating the individuals from the same group, and are colour coded according to language family: blue, Kx’a; red, Tuu; green, Khoe. Modified from Montinaro *et al*. (31), which should be consulted for further details.

In addition to carrying considerable amounts of genetic diversity, Khoisan-speaking populations are also quite diverged from one another, as shown by a deep split between populations residing in the northwestern Kalahari and those from the southeastern Kalahari or South Africa (29,30), respectively. Recent reanalyses of these data together with some new data (31)—providing the most complete geographical coverage of extant Khoisan-speaking groups—that focus solely on genomic segments of Khoisan-related ancestry have demonstrated a tripartite split into northern, central and southern Khoisan-speaking groups [roughly corresponding to Pickrell *et al*.’s (29) northwestern and southwestern and Schlebusch *et al*.’s (30) southern groups, respectively, and also corresponding to groups defined by ecogeographic boundaries in (32); Fig. 2]. It should be noted that these northern, central and southern genetic groupings do not correspond to a previous linguistic classification of southern African Khoisan languages into Northern (Khoisan), Central (Khoisan), Southern (Khoisan) branches (6): the northwestern/northern grouping includes not only the !Xuun and Ju|’hoan, whose languages belong to the Kx’a family, but also the Hai‖om, whose language belongs to the Khoe family; the southeastern/central grouping includes populations speaking languages belonging to all three families, and the southern grouping includes both the pastoralist Nama, whose language belongs to the Khoe family, and the descendants of foragers, the Karretjie and ǂKhomani, whose heritage languages belonged to the Tuu family.

However, there is uncertainty as to when this deep split occurred. Initial studies based on genome-wide single-nucleotide polymorphism (SNP) array data dated the divergence to ~25–35 kya (29–31). The inclusion of genome sequence data from a 2000-year-old hunter-gatherer individual from South Africa pushed back the date of divergence between ‘northern’ and ‘southern’ Khoisan to 156–185 kya (23), which might suggest that the SNP array data underestimate divergence times due to ascertainment bias. Nevertheless, subsequent estimates based on whole genome sequences range from ~30 to ~160 kya (Table 1); different mutation rates can account for some differences in these estimates, but not all, leaving this an open question.

In any event, while this deep divergence between northern, central and southern Khoisan-speaking populations suggests that they must have been isolated from each other for a considerable period of time, there is also evidence for gene flow among Khoisan-speaking groups taking place at a more recent time-scale. This is shown by analyses focussing on genome segments of Khoisan-specific ancestry that show a high correlation of genetic with geographic distances, and a clear signal of isolation by distance (31,33). It is therefore possible that the deep divergence times arise purely as a consequence of long-distance separation in what is actually a gradient of relatedness. However, it is also possible that the signals of isolation by distance reflect more recent processes after initial older divergence events. In particular, it has been suggested that Khoisan-speaking groups were initially split by the prehistoric lake Makgadigadi, with gene flow being reinitiated when the lake dried up around 10 kya (34). One group in particular that shows evidence for admixture are the Naro, who are both geographically (Fig. 1) and genetically (Fig. 2) intermediate between the northwestern/northern and southeastern/central groupings (29,31) and who also show evidence for gene flow from the G|ui and an ethnolinguistically undefined group from Xade in the Central Kalahari Game Reserve (33). In addition, the ǂHoan, who speak a divergent language of the Kx’a family nowadays called ǂ’Amkoe, show only 5% shared ancestry with their linguistic relatives the !Xuun and the Ju|’hoan (33), whereas they are genetically close to the neighbouring Taa (who speak a Tuu language) and the G|ui, whose language belongs to the Khoe family (31) (cf. 34,35). Distinguishing between long-term isolation by distance, versus deep divergence followed by more recent contact, may be possible when more whole genome sequence data become available.

## Admixture with Immigrating Food-Producing Populations

**Figure 3:**
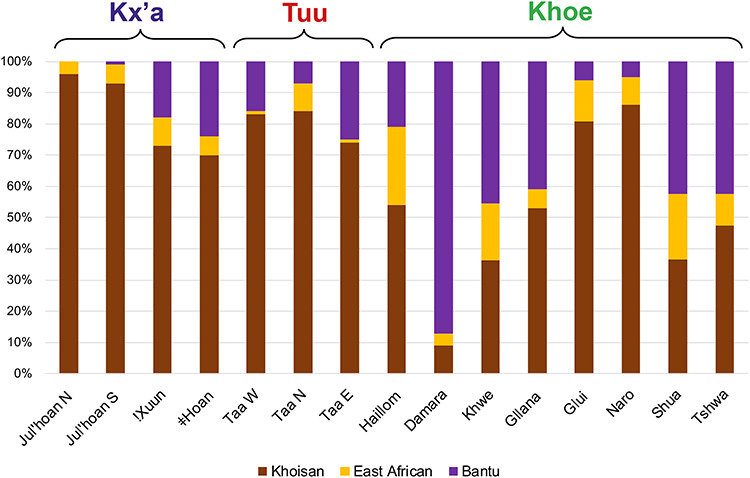
Variable amounts of Khoisan-related, Bantu-related and East African-related ancestries in Khoisan-speaking groups, based on Pickrell *et al*. (36).

In addition to gene flow among Khoisan-speaking groups, these have also undergone variable amounts of admixture from immigrating food-producers (23,36) (Fig. 3). Sheep and goat pastoralists are thought to have immigrated to southern Africa from East Africa a few centuries before the immigration of Iron Age agropastoralists commonly associated with the expansion of Bantu-speaking peoples into large parts of sub-Saharan Africa (37). The presence among southern African populations of the Lactase Persistence variant C-14010 (30,38,39), which is of probable East African origin (40), points towards a demic diffusion of pastoralism into southern Africa. Significantly higher frequencies of this variant in pastoralist populations than in foragers, and in groups speaking languages of the Khoe family than in Tuu- or Kx’a-speaking populations (39), support the hypothesis that the Khoe–Kwadi languages were brought to southern Africa by a migration of pastoralists from East Africa (8). Unexpectedly, however, the formerly Khoe–Kwadi-speaking pastoralist Kwepe from southwestern Angola have only low frequencies of this allele (41), in accordance with their low frequency of the East African Y-chromosome haplogroup E-M293. The spread of pastoralism and the Khoe–Kwadi languages is therefore likely to been a complex process, which might also have involved shift of Bantu-speaking groups to Khoe–Kwadi languages (42). Interestingly, two modern-day forager groups, the G|ui and the Tshwa, show evidence for ongoing positive selection for the C-14010 allele, indicating a possibly recent reversion from a herding way of life to foraging (39). Ancient DNA analyses have provided further direct evidence for admixture with East African pastoralists: a 1200-year-old specimen found in a herder context in the western Cape was shown to have ~40% ancestry related to an early pastoralist from Tanzania and ~60% ancestry related to 2000-year-old South African foragers (14). Two Early Iron Age individuals from Botswana—who are likely to have spoken Bantu languages—confirm the earlier presence of East African pastoralists than Iron Age agropastoralists in the region, since they carry ancestry related to the 1200-year-old admixed herder from the western Cape (15).

The admixture with food-producing populations did not take place at the same time or to the same extent across southern Africa (29,36). Analyses of uniparental data show a strongly sex-biased signal of gene flow in southern Africa, with Khoisan-speaking populations receiving paternal lineages from food-producers, whereas Bantu-speaking groups incorporated mainly Khoisan-related maternal lineages. The intensity of this sex bias increases from North to South, possibly indicating changes in social interactions between immigrating groups and autochthonous peoples over time (35). Such changes in interactions are also implied by the varying levels of Khoisan-related ancestry detectable in modern-day Bantu-speaking populations of southern Africa: populations from Malawi do not show any evidence for Khoisan-related ancestry (14), and populations from southern Mozambique show only low levels of such ancestry [4–5% maximum (43)]. This is in contrast to populations such as the Kgalagadi and Tswana from Botswana with 33–39% and 22–24% Khoisan-related ancestry, respectively (29,36), or the Sotho, Xhosa and Zulu from South Africa with between ~10–24% Khoisan-related ancestry (43,44). Such changes in social interactions between immigrating Iron Age agropastoralists and resident Khoisan-speaking populations might also explain variable patterns of click borrowing in Bantu languages (18,45).

## Ethical Considerations

Indigenous communities are playing an increasingly prominent role in genomics research, going beyond merely providing samples to being fully informed about the results and how they are presented (46,47). Even well-meaning scientists engaged in research on indigenous peoples can fail to appreciate how their scientific statements about their results may be viewed and interpreted by the individuals and communities studied—a prominent example involved a study that sequenced the genomes of four Khoisan-speaking individuals (cf. 48). One outcome of such misunderstandings was the establishment of the San Code of Research Ethics in 2017 (https://www.globalcodeofconduct.org/affiliated-codes/), the first such ethics code by an indigenous African group, and a model for research involving Khoisan-speaking groups. Nonetheless, ethical difficulties continue to arise (49).

## Conclusion

The stereotypical image of Khoisan-speaking peoples as Stone Age hunter-gatherers who have lived in splendid isolation since the dawn of humankind can, without any doubt, be laid to rest. These groups exhibit extensive cultural, linguistic and biological diversity. They harbour more genetic diversity, the earliest divergences and larger effective population sizes than other human populations. They used to be more widespread in former times, are likely to have engaged in long-distance migrations and they have both influenced and been influenced by at least two migrations, an earlier migration of pastoralists from eastern Africa and a later migration of agropastoralists associated with the spread of Bantu languages. Understanding the complex genomic history and structure of Khoisan-speaking populations has important implications not only for their individual histories and the history of humans in general, but also for potential variation in disease susceptibility (cf. 50,51). There is a clear need for further whole genome sequence studies of Khoisan-speaking groups, in order to achieve these goals.
